# A child with intellectual disability and dysmorphism due to complex ring chromosome 6: identification of molecular mechanism with review of literature

**DOI:** 10.1186/s13052-018-0571-0

**Published:** 2018-10-11

**Authors:** Frenny Sheth, Thomas Liehr, Viraj Shah, Hillary Shah, Stuti Tewari, Dhaval Solanki, Sunil Trivedi, Jayesh Sheth

**Affiliations:** 1 FRIGE’s Institute of Human Genetics, FRIGE House, Jodhpur Gam Road, Satellite, Ahmedabad, 380009 India; 2 University Clinic Jena, Institute of Human Genetics, Am Klinikum 1, 07747 Jena, Germany; 3 Mantra Child Neurology & Epilepsy Hospital, 3rd floor, Oarnate complex, Kalubha road, Kalanala, Bhavanagar, 364001 India

**Keywords:** Molecular karyotyping, Molecular cytogenetics, Haploinsufficiency, Ring chromosome, R(6)

## Abstract

**Background:**

Ring chromosome 6 (r(6)) is a rare disorder that mainly occurs as a ‘de novo’ event. Nonetheless, a wide phenotypic spectrum has been reported in r(6) cases, depending on breakpoints, size of involved region, copy number alterations and mosaicism of cells with r(6) and/or monosomy 6 due to loss of r(6).

**Case presentation:**

An 11-year-old male was referred with developmental delay, intellectual disability and microcephaly. Physical examination revealed additionally short stature and multiple facial dysmorphisms. Banding cytogenetic studies revealed a karyotype of mos 46,XY,r(6)(p25.3q27)[54]/45,XY,-6[13]/46,XY,r(6)(::p25.3→q27::p25.3→q27::)[13]/46,XY[6]/47,XY,r(6)(p25.3q27)×2[2]dn. Additionally, molecular karyotyping and molecular cytogenetics confirmed the breakpoints and characterized a 1.3 Mb contiguous duplication at 6p25.3.

**Conclusion:**

The present study has accurately identified copy number alterations caused by ring chromosome formation. A review of the literature suggests that hemizygous expression of *TBP* gene in 6q27~qter, is likely to be the underlying cause of the phenotype. The phenotypic correlation and clinical severity in r(6) cases continue to remain widely diverse in spite of numerous reports of genomic variations.

## Background

Ring chromosome 6 (r(6)) is an exceedingly rare disorder, first described in 1973 by Moore et al. [1]. Since then, >30 patients with this condition have been reported [2]. Formation of ring chromosomes usually occurs due to breaks in the terminal portions of both the chromosome arms, followed by the fusion of broken ends. Alternatively, they can be formed by the union of subtelomeric sequences or telomere-telomere fusion with no deletion, resulting in a complete ring chromosome. Such complete rings without apparent significant loss of genetic material have been described in individuals with normal phenotypes [3]. Other mechanisms for ring chromosome formation, like terminal deletions, and/ or contiguous inverted duplication due to an inv-dup-del rearrangement/s have also been proposed in the literature [4–6], also recently chromothripsis has been attributed as a possible reason for such cases [7].

Even though being rare, a wide spectrum of phenotypic variability is observed in all cases of autosomal ring chromosomes. Congenital heart defects, intellectual disability, microcephaly, facial dysmorphism, failure to thrive and various abnormalities in the ocular, auditory and central nervous systems are frequently detected [8].

In this study, a mosaic r(6) in a clinically abnormal, 11-year-old male was characterized in detail and compared with cases from the literature to share some new insights into the genotype-phenotype correlations.

## Case presentation

An 11-year-old male was referred for further diagnostics due to developmental delay, intellectual disability and microcephaly. He was the first child born to apparently healthy non-consanguineous parents. The mother had an uneventful pregnancy with no history of prenatal exposure to alcohol, drug or tobacco. Though his siblings (7-year brother and 3 years old sister) were phenotypically normal, his paternal cousin-sister was microcephalic and mentally challenged. No investigations were carried out in the affected cousin-sister.

**Fig. 1:**
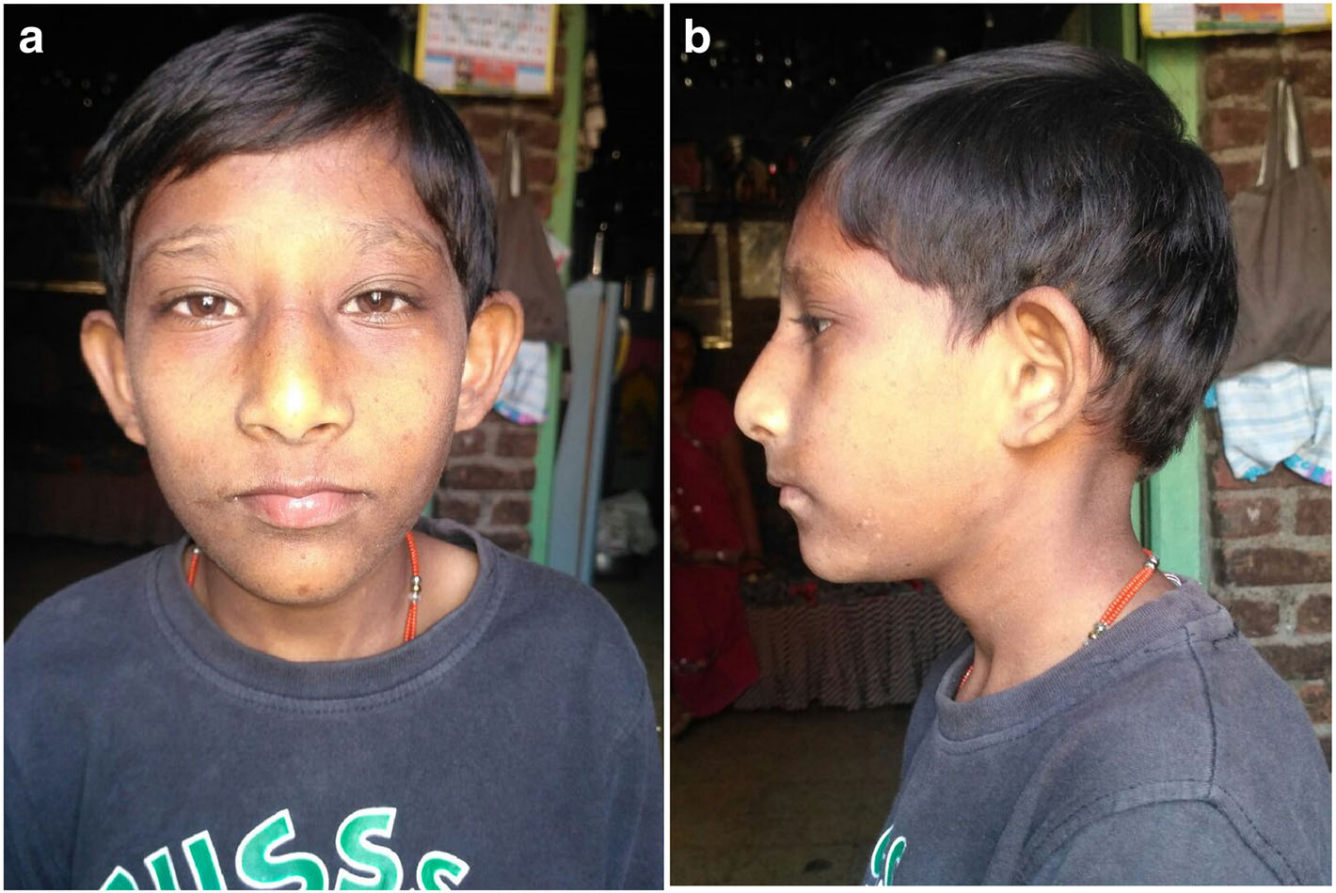
**a** and **b** Facial phenotype of the proband

**Fig. 2:**
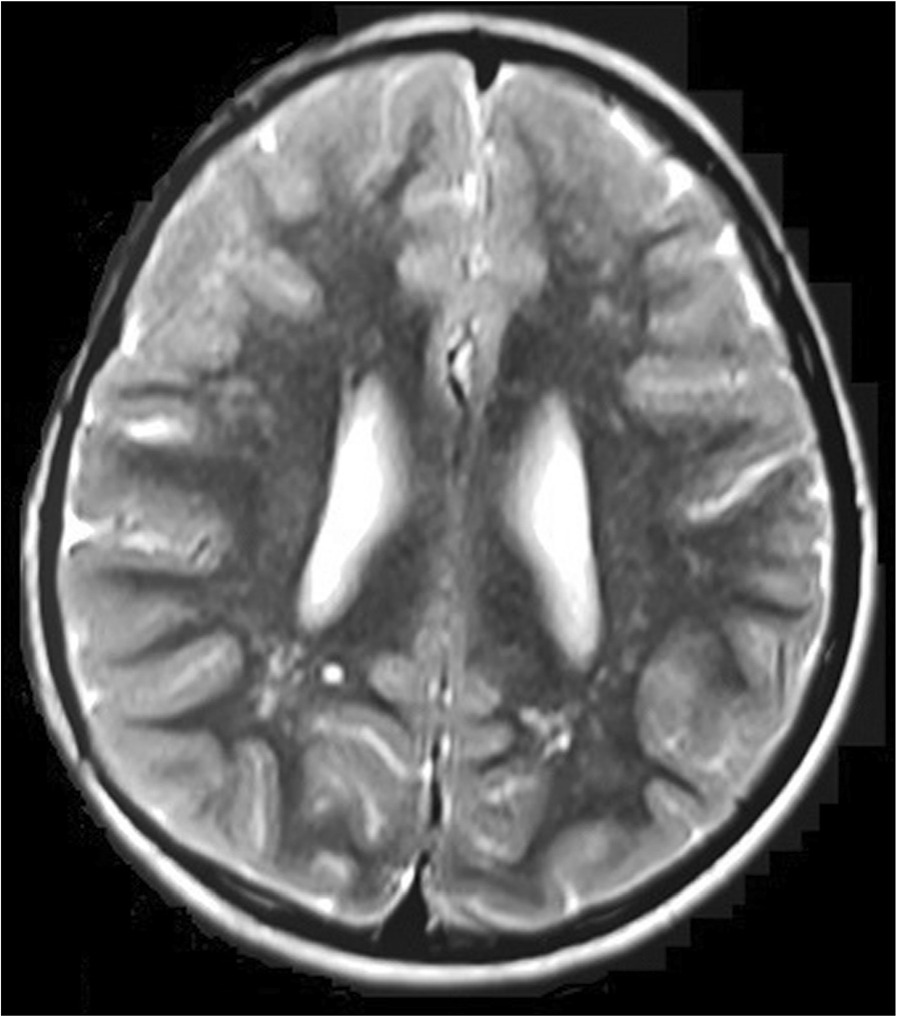
MRI picture depicting bilateral frontal and parietal lobe white matter changes

The proband was born by normal vaginal delivery. The birth weight was 1.5 kg and head circumference was 33 cm. Apgar score at birth was within the normal range. The patient was sitting without support at around 1.5 years. He could stand with support by 2 years and independent walking at 2.5 years. His speech development was delayed. He was not able to speak sentences and could not achieve proper bowel and bladder control even at the time of presentation (11 years). His height and weight were 108 cm and 14.5 kg respectively (below 10th centile); head circumference (OFC; Occipital Frontal Circumference) was 42 cm (below 3rd centile). The proband portrayed short stature and microcephaly with developmental delay. The facial dysmorphism showed long face, small chin, large protruding ears, slightly upturned eyes, sparse eyebrows, large bulbous nose and thin upper lip (Fig. 1a and b). Speech delay, penile chordee and sacral dimple were also noted during physical examination. His respiratory, cardiovascular and abdominal examinations were unremarkable. CNS examination showed intellectual disability, delayed language development and hyperactivity. His speech and cognitive development was more delayed than his motor milestones, and academic performance was very poor. His serum TSH (thyroid stimulating hormone) and GH (growth hormone) levels were normal. Magnetic resonance imaging (MRI; performed at an age of 10 years) showed demyelination in both frontal and parietal lobes (Fig. 2). Ultrasonography of abdomen and pelvis were normal, as were echocardiogram and ophthalmologic examinations.

**Fig. 3:**
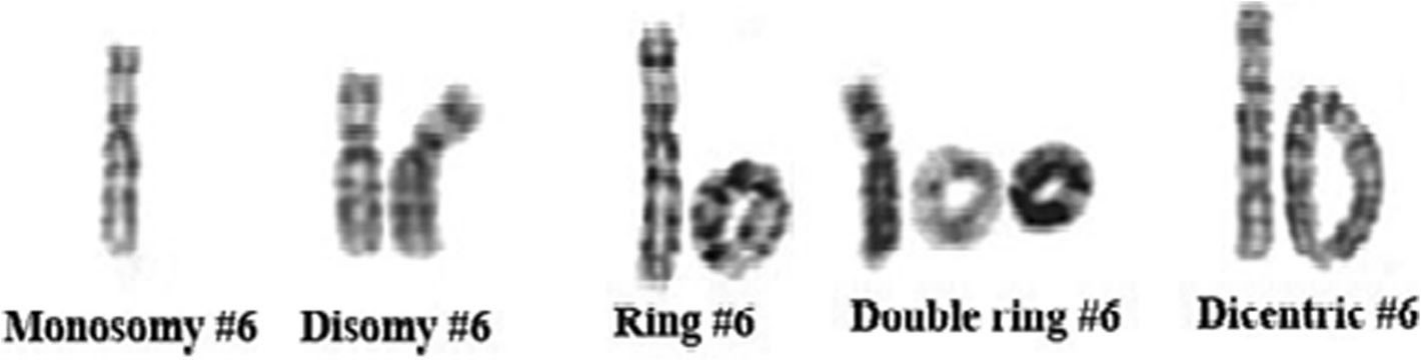
Partial G-banded karyotype showing various patterns of r(6) in multiple cell lines as mos 46,XY,r(6)(p25.3q27)[54]/45,XY,-6[13]/46,XY,r(6)(::p25.3→q27::p25.3→q27::)[13]/46,XY[6]/47,XY,r(6)(p25.3q27)×2[2]dn

Sample collection and written informed consent was obtained according to the need of the institutional ethics committee in accordance with Helsinki declaration. Chromosome analysis of the patient was performed with 72 h lymphocyte culture and standard GTG-banding. The karyotype was interpreted according to the International System for Human Cytogenetic Nomenclature (ISCN 2016) [9] as mos 46,XY,r(6)(p25.3q27)[54]/45,XY,-6[13]/46,XY,r(6)(::p25.3→q27::p25.3→q27::)[13]/46,XY[6]/47,XY,r(6)(p25.3q27)×2[2]dn (Fig. 3).

**Fig. 4:**
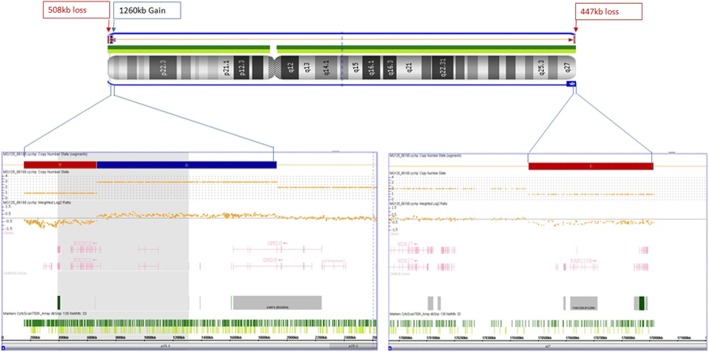
Chromosomal microarray showed subtelomeric deletions and a 1.3 Mb duplication in 6p25.3

**Fig. 5:**
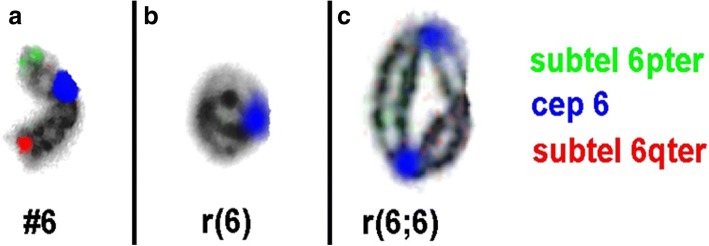
FISH study showed **a**) 3 signals (green, blue and red) that confirm an intact chromosome 6. **b**) single signal (blue) on r(6) confirm subtelomeric deletions at both the arms. **c**) Two signals (blue) confirm two centromeres present in a dicentric ring

Karyotype of the parents was normal, confirming a ‘de novo’ origin of the r(6). Further analysis was carried out by molecular karyotyping using Affymetrix CytoScan™ 750 K array. Data was analyzed using Chromosome Analysis Suite (ChAS) and revealed: arr [GRCh37] 6pterp25.3(156,974_665,234)x1,6p25.3(668,700_1,929,528)x3,6q27qter(170,466,513_170,914,297)×1. In other words, there was a partial terminal monosomy of overall 508 kb in 6p25.3 followed by a 1.3 Mb contiguous duplication in 6p25.3 and 448 kb terminal deletion in 6q27 (Fig. 4). These results were confirmed by fluorescence in situ hybridization (FISH) using commercially available subtelomeric probes for 6pter and 6qter. The duplicated region in 6p25.3 could not be confirmed by another method due to lack of corresponding locus-specific probes. The dicentric nature of double ring was thus established by a centromeric probe (Fig. 5).

## Discussion and conclusions

Ring chromosome 6 is a rare event, that generally occurs as a ‘de novo’ event. Inheritance of ring chromosomes is quite infrequent since it is unstable during cell division with a possible loss of the ring during meiosis. Moreover, ring chromosome carriers may be infertile due to the chromosomal alteration.

Postnatal growth failure and microcephaly are the leading features of any autosomal ring chromosome; also they tend to show dynamic mosaicism, as observed in the present case. Patients with r(6) syndrome frequently present with growth retardation, facial dysmorphism and microcephaly. The additional clinical features of the present case can most likely be attributed to the cryptic duplication event in the r(6).

Major features associated either with 6p or 6q terminal deletions include defects of the anterior eye-chamber development, hearing loss, heart malformations, hypertelorism, mid-face hypoplasia, low set ears, developmental delay, intellectual disability, hypotonia, seizures, facial dysmorphism, and short neck [10, 11]. Most of these findings are analogous to that noted in the proband under report which suggests that the phenotypic variability and severity may partially be attributed to existence and instability of the ring. Several mechanisms for such instability are proposed in the literature, without plausible explanation [3, 12]. Further, the severity of the phenotype is hypothesized to be related to the size of the deletion. Microcephaly, facial dysmorphism and cardiac abnormalities seem to be more prevalent in the patients with single cell line anomaly; conversely, growth retardation, intellectual disability and demyelination of frontal and parietal lobes were additional anomalies that were more frequent with multiple cell lines.

**Fig. 6:**
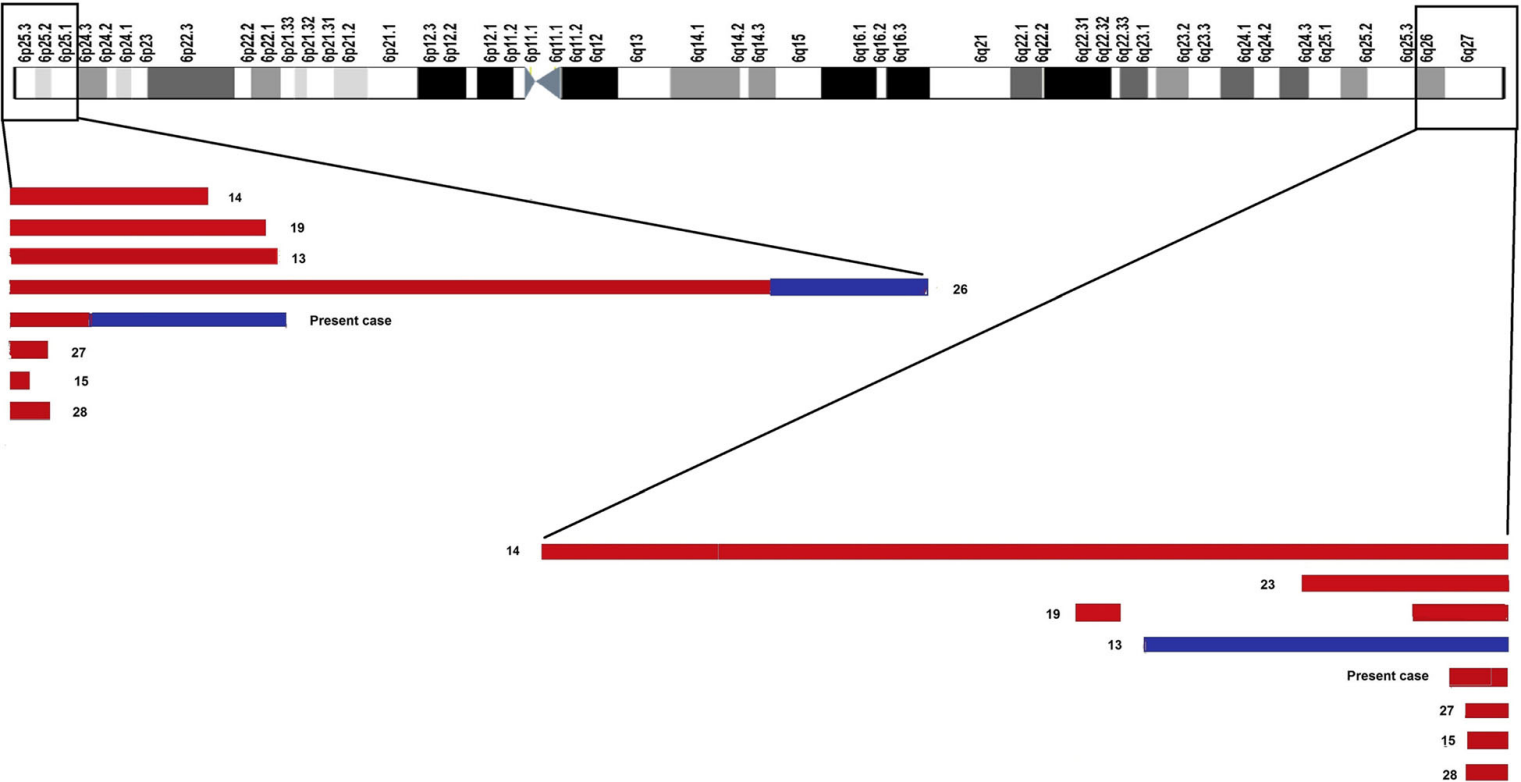
Schematic representation of chromosome 6 reveal deleted and/or duplicated genomic regions with previously reported cases with r(6). Breakpoint distribution is based on the molecular cytogenetic data seen in cases having r(6). The deleted (red) and duplicated regions (blue) are shown according to a scale of 1Mb = 3.2 cm. Numerical number mentioned by bars indicate the correcponding references [26–28]

To establish genotype/phenotype correlations for r(6), we compared the clinical features of the case under report to previously reported cases of r(6) (Table 1; Fig. 6) and explored the deleted and duplicated regions for morbid genes, using OMIM database and DECIPHER (https://decipher.sanger.ac.uk/).

**Table 1:** Comparative clinical features and cytogenetic results in various cases of ring chromosome 6

	Single cell line (one of the chromosome #6 has been replaced by a ring chromosome 6)								Mosaicism (structural and numerical variants of ring chromosome #6 are present in various cell lines in different proportions)								
									2 cell lines			3 cell lines	4 cell lines		5 cell lines		
Publications	Pace et al. [13]	Ciocca et al. [14]	Ahzad et al. [15]	Walker et al. [16]	Kini et al. [17]	Fried et al. [18]	Zhang et al. [19]	Fraction	Hurd et al. [20]	Lee et al. [8]	Andrieux et al. [21]	Kara et al. [22]	Nishigaki et al. [23]	Hockner et al. [24]	Nishi et al. [25]	Present case	Fraction
Karyotype	Not available	46,XX,r(6)(p25q27)	46,XY,r(6)(p25q27)	46,XY,r(6)(p25q27)	Not available	Not available	46,XY,r(6)(p25q27).ish r(6)(p25.1q27)		47, XY, +r [17]/48,XY, +rx2 [3] and second case 47, XY, +mar[12]/46,XY[3]	46, XY, r(6)(p25q27)/46, XY, dic r(6;6)(p25q27;p25q27)	46,XY,r(6)(p25q27)/45,XY-6	mos46,XY,r(6)(p24;q26),del(6)(q27) [30]/46,XY,del(6)(q27) [20]	mos 46,XX,r(6)(p25q27)[67]/45,XX,-6[25]/46,XX,dic r(6:6)(p25q27:p25q27)[6]/47,XX,r(6)(p25q27)× 2[2]	mos 46,XX,r(6)(p25.3q27)[16]/45,XX,-6[2]/46,XX,dic r(6:6)(p25.3q27:p25.3q27)[2]/47,XX,r(6)(p25.3q27) × 2[1]	Not available	mos 46,XY,r(6)(p25.3q27)[54]/45,XY,-6[13]/46,XY,r(6)(::p25.3→q27::p25.3→q27::)[13]/46,XY[6]/47,XY,r(6)(p25.3q27)×2[2]dn	
Clinical features																	
Microcephaly	√		√	√	√	√	√	6/7		√	√	√	√	√	√	√	7/8
Facial dysmorphism	√	√	√		√	√		5/7	√	√	√	√	√	√		√	7/8
Glaucoma							√	1/7									
Hearing loss	√			√			√	3/7	√								1/8
Tachypnea								–		√							1/8
Speech delay								–	√				√			√	3/8
White matter abnormalities in brain								–			√		√			√	3/8
Developmental delay	√	√		√				3/7	√				√	√		√	4/8
Growth retardation	√				√		√	3/7					√	√	√	√	4/8
Intellectual disability			√			√		2/7				√		√	√	√	4/8
Scoliosis	√							1/7						√			1/8
Cardiac anomalies (ASD,CHD,PDA)	√	√	√					3/7		√							1/8
Abnormalities in the digits (brachydactyly/clinodactyly/bilateral syndactyly)	√		√				√	3/7		√	√	√	√				4/8
Single palmar crease			√					1/7									–
Penile chordee								–								√	1/8
Sacral dimple								–								√	1/8
Abnormalities in the foot	√							1/7		√							1/8
Complex bone disorders								–	√ (2)								2/8
Dry skin	√							1/7									–
seizures				√	√		√	3/7	√ (2)				√				3/8
Umbilical hernia			√					1/7									–

*ASD* atrial septal defect, C*HD* congenital heart defect, *PDA* patent ductus arteriosus

The deleted 508 kb subtelomeric region from 6pter to 6p25.3 contains 4 annotated genes: *DUSP22, IRF4, EXOC2, HUS1B*; *IRF4* (*601900) being the only OMIM morbid gene. Interferon regulatory factor 4 (*IRF4*) is a transcription factor essential for the development of T helper 2 (Th2), Th17 and Th9 cells, whose allelic variant influences variation in skin/hair/eye pigmentation 8. Dysregulation of *IRF4* is associated with lymphoid malignancies [29].

The duplicated segment of 1.3 Mb at 6p25.3 was larger in size and contained more relevant OMIM genes-three copies each of *EXOC2, LOC285768, FOXQ1, FOXF2, FOXC1, GMDS, FOXCUT*; *FOXC1* being the only morbid gene amongst them. Mutations in Forkhead box C1; (*FOXC1*; *601090) has been associated with phenotypes like Anterior segment dysgenesis 3 and Axenfeld Reigen syndrome type 3. Nishimura et al. [30] in their study demonstrated that haploinsufficiency in addition to the mutations as well as increased gene dosage may cause anterior chamber defects of the eye. Later, Lehman et al. [31] reported an association between interstitial 6p25 duplications involving *FOXC1* gene and ocular developmental abnormalities and glaucoma. Ophthalmologic examination in the patient under report did not reveal any abnormality and possibly point towards a less likely role of an extra copy of *FOXC1* alone in the ophthalmological manifestations in such patients.

Apart from ocular manifestations, mutations and haploinsufficiency of *FOXC1* gene have also been speculated to be implicated in cardiac abnormalities [2, 32]. However, there seem to be no reports to suggest for duplication. The patient under report had normal echocardiography findings. There is a paucity of data on the pathological manifestations of distal 6p duplication. Qi et al. elaborated on the same in 2015 study and concluded that copy number gain of this particular region is likely benign or triploinsensitive [33].

A terminal deletion (448 kb) was detected (from 6q27 to 6qter) that encompass *LOC154449, DLL1, FAM120B, MIR4644, PSMB1, TBP, PDCD2* OMIM annotated genes. Amongst them, TATA-binding protein (TBP), a general transcription factor associates with aggregates in several polyglutamine disorders; is the only gene with a pathogenic potential [34]. A reasonable number of studies have postulated *TBP* as a potential candidate gene responsible for the phenotype of patients with subtelomeric 6q deletions, irrespective of the size of terminal deletion [35, 36]. Moreover, a study [37] has reported a ‘de novo’ missense mutation in the chromosome 6 open reading frame 70 (*C6orf70*) gene in 1/14 (7.1%) patient with periventricular nodular heterotopia, developmental delay and epilepsy through whole exome sequencing. In addition, the authors silenced C6orf70 and two additional genes (*phf10 and Dll1*) in the developing rat neocortex and suggested that C6orf70 plays a major role in the control of neuronal migration and its haploinsufficiency or mutation causes periventricular heterotropia.

Developmental delay, intellectual disability, dysmorphism, seizures, dimpling of elbows and knees are some of the main features noted in such patients. Most of the above mentioned clinical features were also seen in our patient, raising a strong possibility pointing towards the hemizygous expression of *TBP* might be implicated in the causation of the phenotype. Apart from these, the developmental delay, growth stunting and mild facial dysmorphism observed in the proband can also be attributed partially to the mitotic instability of the ring chromosome as the source of continuous production of secondary aneuploid cells [3, 25]. There is still a dearth of literature on ring chromosome 6 and therefore, further studies are advocated to understand the role of genes influencing such genotype-phenotypic presentations.

## Abbreviations

DLL1: Delta like canonical notch ligand 1
DUSP22: Dual specificity phosphatase 22
EXOC2: Exocyst complex component 2
FAM120B: Family with sequence similarity 120B
FISH: Fluorescence in situ hybridization
FOXC1: Forkheadbox C1
FOXCUT: FOXC1 upstream transcript
FOXF2: Forkheadbox F2
FOXQ1: Forkhead box protein Q1
GMDS: GDP-mannose 4,6-dehydratase
HUS1B: HUS1 checkpoint clamp component B
IRF4: Interferon regulatory factor 4
ISCN: International System for Human Cytogenetic Nomenclature
MIR4644: MicroRNA 4644
PDCD2: Programmed cell death 2
PSMB1: Proteasome subunit beta type-1
r(6): Ring chromosome 6
SNP: Single nucleotide polymorphism
TBP: TATA-binding protein
